# Effect of alirocumab on individuals with type 2 diabetes, high triglycerides, and low high-density lipoprotein cholesterol

**DOI:** 10.1186/s12933-020-0991-1

**Published:** 2020-02-08

**Authors:** Helen M. Colhoun, Lawrence A. Leiter, Dirk Müller-Wieland, Bertrand Cariou, Kausik K. Ray, Francisco J. Tinahones, Catherine Domenger, Alexia Letierce, Marc Israel, Rita Samuel, Stefano Del Prato

**Affiliations:** 1 The Institute of Genetics and Molecular Medicine, University of Edinburgh, Western General Hospital, Crewe Road, Edinburgh, EH4 2XU UK; 2 Li Ka Shing Knowledge Institute, St. Michael’s Hospital, University of Toronto, Toronto, ON Canada; 3 Department of Internal Medicine I, University Hospital Aachen, Aachen, Germany; 4 l’institut du thorax, CHU Nantes, INSERM, CNRS, UNIV Nantes, Nantes, France; 5 Imperial Centre for Cardiovascular Disease Prevention, Department of Primary Care and Public Health, Imperial College, London, UK; 6 Department of Clinical Endocrinology and Nutrition (IBIMA), Hospital Virgen de la Victoria, University of Málaga, CIBER Fisiopatología de la Obesidad y Nutrición (CIBERobn), Instituto de Salud Carlos III, Málaga, Spain; 7 Sanofi, Gentilly, France; 8 Biostatistics and Programming Department, Sanofi, Chilly-Mazarin, France; 9 Regeneron Pharmaceuticals, Inc, Tarrytown, NY USA; 10 Department of Clinical and Experimental Medicine, University of Pisa, Pisa, Italy

**Keywords:** Alirocumab, PCSK9, Diabetes mellitus, Non-HDL-C, HDL-C, Triglycerides, ODYSSEY, DM-DYSLIPIDEMIA, Type 2 diabetes, Usual care

## Abstract

**Background:**

Mixed dyslipidemia [elevated non-high-density lipoprotein cholesterol (non-HDL-C) and triglycerides (TGs), and decreased HDL-C] is common in type 2 diabetes mellitus (T2DM) and is associated with increased cardiovascular risk. Non-HDL-C and apolipoprotein B (ApoB) are the preferred therapeutic targets for mixed dyslipidemia. Alirocumab is a monoclonal antibody to proprotein convertase subtilisin/kexin type 9 (PCSK9) that effectively reduces low-density lipoprotein cholesterol (LDL-C), non-HDL-C, ApoB, and lipoprotein(a) (Lp[a]), and is well-tolerated in individuals with T2DM.

**Methods:**

The previously reported open-label ODYSSEY DM-DYSLIPIDEMIA trial data demonstrated the effects of alirocumab on individuals with non‐HDL-C ≥ 100 mg/dL and TGs ≥ 150 and < 500 mg/dL receiving stable maximally tolerated statin (n = 413). This post hoc subgroup analysis of the primary trial investigated the effects of alirocumab [75 mg every 2 weeks (Q2W) with possible increase to 150 mg Q2W at Week 12] versus usual care [ezetimibe, fenofibrate, or no additional lipid-lowering therapy (LLT)] on non-HDL-C and other lipids in individuals with T2DM and baseline TGs ≥ 200 mg/dL and HDL-C < 40 mg/dL (men) or < 50 mg/dL (women).

**Results:**

Alirocumab significantly reduced non-HDL-C [LS mean difference (standard error (SE)), − 35.0% (3.9)], ApoB [LS mean difference (SE), − 34.7% (3.6)], LDL-C [LS mean difference (SE), − 47.3% (5.2)], LDL particle number [LS mean difference (SE), − 40.8% (4.1)], and Lp(a) [LS mean difference (SE), − 29.9% (5.4)] versus usual care from baseline to Week 24 (all *P* < 0.0001). Results were similar for alirocumab versus usual care. TG reductions were similar between alirocumab and usual care (no significant difference), but greater with fenofibrate versus alirocumab (*P *= 0.3371). Overall, alirocumab significantly increased HDL-C versus usual care [LS mean difference (SE), 7.9% (3.6); *P* < 0.05], although differences with alirocumab versus ezetimibe or fenofibrate were non-significant. Most individuals receiving alirocumab achieved ApoB < 80 mg/dL (67.9%) and non-HDL-C < 100 mg/dL (60.9%). Adverse event frequency was similar between alirocumab (67.2%) and usual care (70.7%). Additionally, no clinically relevant effect of alirocumab on change in glycemic parameters or use of antihyperglycemic agents was observed.

**Conclusions:**

Alirocumab is an effective therapeutic option for individuals with T2DM, TGs ≥ 200 mg/dL, and HDL-C < 40 mg/dL (men) or < 50 mg/dL (women). Atherogenic lipid (ApoB and non-HDL) reductions were greater with alirocumab than ezetimibe, fenofibrate, or no LLT. Consistent with previous studies, alirocumab was generally well tolerated.

*Trial registration* Clinicaltrials.gov, NCT02642159. Registered December 24, 2015, https://clinicaltrials.gov/ct2/show/NCT02642159

## Background

Individuals with type 2 diabetes mellitus (T2DM) are at increased risk of atherosclerotic cardiovascular disease (ASCVD) [1]. Mixed dyslipidemia, i.e. elevated plasma triglycerides (TGs), TG-rich lipoprotein (TRL) and TRL cholesterol (TRL-C) levels and decreased levels of high-density lipoprotein cholesterol (HDL-C) [2], is a major contributor to ASCVD risk in individuals with diabetes [3, 4]. Individuals with mixed dyslipidemia may also have an elevated number of small, dense low-density lipoprotein (LDL) particles [2, 5], as reflected by higher levels of ApoB-100 (ApoB); however, these individuals may not necessarily have elevated LDL cholesterol (LDL-C) levels [6].

ApoB-containing lipoproteins have been demonstrated to be directly associated with the risk of coronary heart disease [7], as indicated by the reduction in cardiovascular risk associated with statin therapy, proportional with the reduction in ApoB [8]. In addition, when LDL-C and ApoB are discordant, as commonly occurs in insulin-resistant states [9], non-HDL-C and ApoB are considered to be stronger predictors of cardiovascular risk than LDL-C [2, 10, 11]. TGs are not a specific therapeutic target in cardiovascular disease; however, when TGs are 200–499 mg/dL, the primary targets of lipid therapy are non-HDL-C and LDL-C [2]. The National Lipid Association recommends non-HDL-C targets of < 130 mg/dL for individuals at high ASCVD risk and < 100 mg/dL for those at very-high ASCVD risk, and ApoB targets of < 90 mg/dL for primary prevention, and < 80 mg/dL for those with very-high cardiovascular risk [2]. The American Association of Clinical Endocrinologists recommends targets of < 100 mg/dL for non-HDL-C and < 80 mg/dL for ApoB in individuals at very-high cardiovascular risk, and targets of < 80 mg/dL for non-HDL-C and < 70 mg/dL for ApoB for individuals at extreme risk [12].

Alirocumab is a monoclonal antibody that binds to circulating proprotein convertase subtilisin/kexin type 9 (PCSK9), which was previously investigated in individuals with mixed dyslipidemia in the ODYSSEY DM-DYSLIPIDEMIA trial [13, 14]. This trial was a Phase 3, randomized, open-label, parallel group, multinational study (NCT02642159) that compared alirocumab with usual care [ezetimibe, fenofibrate, omega-3, niacin, and no additional lipid-lowering therapy (LLT)] in adults with T2DM and mixed dyslipidemia (non‐HDL-C ≥ 100 mg/dL and TGs ≥ 150 and < 500 mg/dL) receiving stable maximally tolerated statin dose (n = 413) [13]. The trial showed that the primary endpoint of reduction in non-HDL-C with alirocumab was superior to usual care overall (mean difference of − 32.5% vs usual care at Week 24; *P* < 0.0001) and versus fenofibrate (mean difference of − 33.3% vs fenofibrate at Week 24; *P* < 0.0001), and that alirocumab was generally well tolerated [14].

The aim of this post hoc analysis of the DM-DYSLIPIDEMIA study was to focus on a higher risk and more difficult to treat subpopulation compared with the primary trial population, and provide analyses of other lipids beyond the primary endpoint.

## Methods

### Primary analysis

Detailed methods of the DM-DYSLIPIDEMIA study have been reported previously [13]. Briefly, individuals were randomized 2:1 to receive alirocumab or usual care for 24 weeks. All individuals were receiving maximally tolerated statin. Usual care included addition of ezetimibe, fenofibrate, no additional LLT, omega-3 fatty acid, or nicotinic acid. Randomization was stratified by the investigator’s choice of usual care therapy, which was prespecified prior to randomization.

The primary analysis included adults with T2DM and mixed dyslipidemia (non‐HDL-C ≥ 100 mg/dL; TGs ≥ 150 and < 500 mg/dL) receiving stable maximally tolerated statin dose for at least 4 weeks prior to screening, without other LLT, and who had a documented history of ASCVD or at least one additional cardiovascular risk factor, and glycated hemoglobin (HbA1c) < 9.0%. The maximally tolerated dose of statin was based on investigator judgement. Individuals with documented statin intolerance and therefore not receiving statin therapy could also be enrolled.

### Post hoc subgroup analysis

In this post hoc subgroup analysis, the effect of alirocumab versus usual care on non-HDL-C and other lipids was investigated in a subgroup of individuals with baseline levels of non-HDL-C ≥ 100 mg/dL, TGs ≥ 200 mg/dL, and HDL-C < 40 mg/dL (men) or < 50 mg/dL (women). The thresholds for TGs and HDL-C in this analysis reflect those used in previous analyses of the effects of fenofibrate on cardiovascular events in the ACCORD (TGs ≥ 204 mg/dL, HDL-C ≤ 34 mg/dL) [15] and FIELD trials [TGs ≥ 150.6 mg/dL, HDL-C ≤ 39.8 mg/dL (men) or ≤ 49.9 mg/dL (women)] [16], and icosapent-ethyl in the amended REDUCE-IT trial protocol [TGs 200–499 mg/dL and HDL-C ≤ 40 mg/dL (men) or ≤ 50 mg/dL (women)] [17].

This analysis provides an overall comparison of alirocumab versus usual care (ezetimibe, fenofibrate, no additional LLT, omega-3 fatty acid, and nicotinic acid), and separate analyses of alirocumab versus ezetimibe, fenofibrate, and no LLT. Due to low participant numbers, nicotinic acid and omega-3 fatty acid strata were not compared separately to alirocumab.

### Endpoint

The primary efficacy endpoint of the DM-DYSLIPIDEMIA study was the percentage change in non-HDL-C from baseline to Week 24. In this analysis, percentage change from baseline in LDL-C (measured by beta-quantification), non-HDL-C, ApoB, LDL particle number, Lp(a), TGs, TRL-C (i.e. non-HDL-C minus LDL-C), and HDL-C with alirocumab and usual care at Week 24 was analyzed in the intention-to-treat population); additionally, estimates for alirocumab versus ezetimibe, fenofibrate, and no LLT differences were derived from the same model with appropriate contrasts.

### Statistical analyses

For LDL-C, non-HDL-C, ApoB, LDL particle number, and HDL-C, least squares (LS) mean difference [standard error (SE)] with alirocumab versus usual care was analyzed by a mixed-effect model with repeat measurements to manage missing data. For Lp(a) and TGs, which are not normally distributed, a combined estimate for adjusted mean difference (SE) with alirocumab versus usual care was calculated by using multiple imputation to manage missing data, followed by robust regression. The combined estimates for proportions (%) of individuals reaching ApoB < 80 mg/dL at Week 24 and non-HDL-C < 100 mg/dL at Week 24 were obtained by using multiple imputation to manage missing data, followed by logistic regression. The safety analysis was descriptive and based on the safety population and was analyzed according to the treatment group (alirocumab or usual care).

## Results

**Fig. 1:**
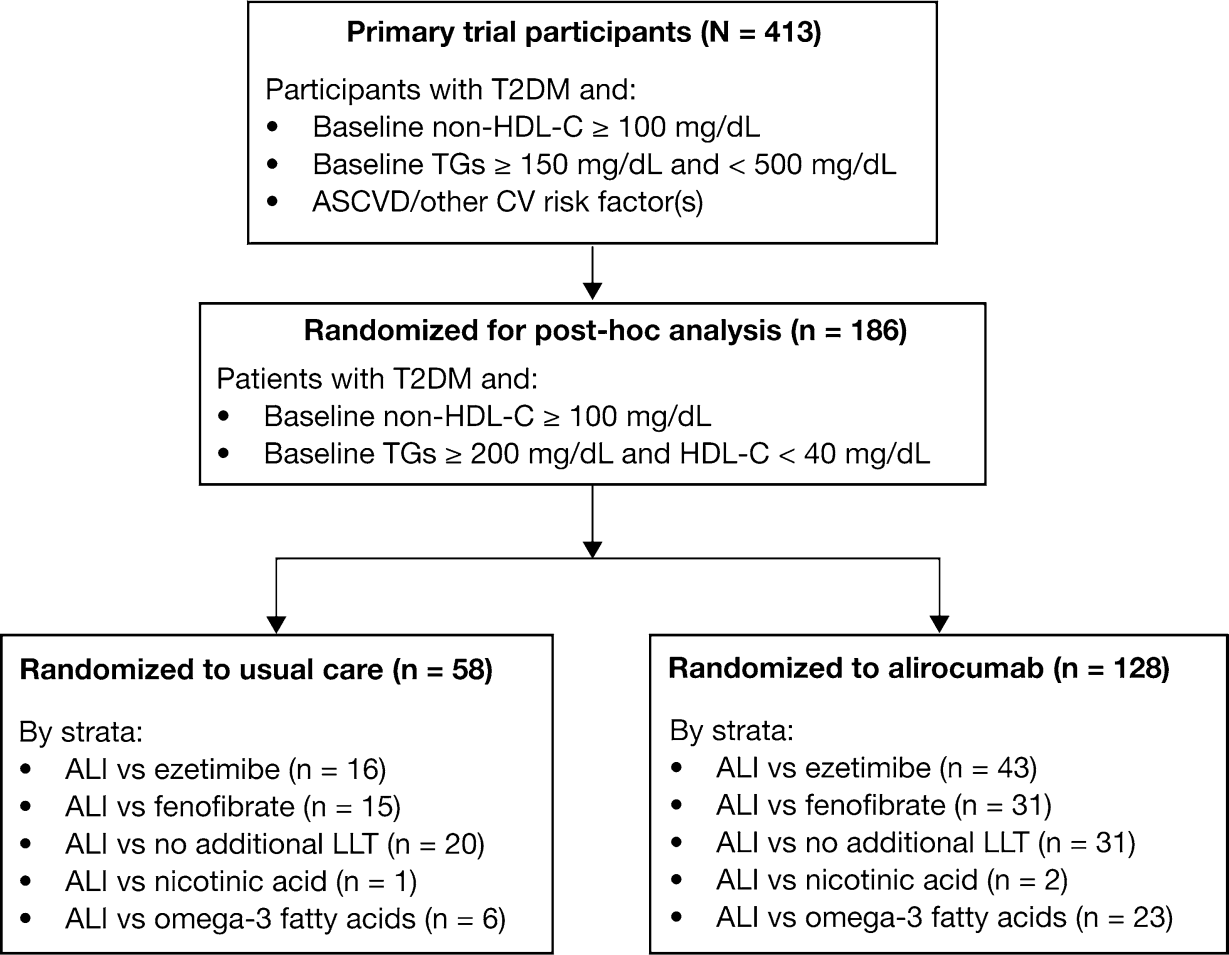
Patient flow chart for the ODYSSEY DM-DYSLIPIDEMIA post hoc analysis. ALI, alirocumab; ASCVD, atherosclerotic cardiovascular disease; CV, cardiovascular; HDL-C, non-high-density lipoprotein cholesterol; LLT, lipid-lowering therapy; TG, triglyceride; T2DM, type 2 diabetes mellitus

Of the 413 individuals included in the primary DM-DYSLIPIDEMIA study, this post hoc analysis included 186 individuals with TGs ≥ 200 mg/dL and HDL-C < 40 mg/dL (men) or < 50 mg/dL (women), randomized to alirocumab (n = 128) or usual care (n = 58). Figure 1 provides the patient flow chart for the ODYSSEY DM-DYSLIPIDEMIA post hoc analysis, showing the number of individuals randomized to each usual care stratum. Separate analyses were conducted for alirocumab versus ezetimibe (n = 16), fenofibrate (n = 15), and no LLT (n = 20). Due to low participant numbers, nicotinic acid (n = 1) and omega-3 fatty acid (n = 6) strata were not compared separately to alirocumab.

Baseline characteristics were generally similar across treatment groups (Table 1). Similar proportions of individuals in the alirocumab and usual care groups had ASCVD (defined as coronary heart disease, peripheral arterial disease, or ischemic stroke; 36.7% vs 41.4%) and were receiving insulin (40.6% vs 44.8%) at baseline. Mean (standard deviation [SD]) HbA1c at baseline was also similar between groups [7.0% (0.9) in the alirocumab group and 7.3% (0.8) in the usual care group].

**Table 1:** Baseline demographics (ITT population)

	Alirocumab (n = 128)	Usual care^a^ (n = 58)
Age, years, mean (SD)	62.1 (9.5)	63.4 (9.0)
Male, n (%)	65 (50.8)	28 (48.3)
Race, n (%)		
American Indian or Alaska Native	3 (2.3)	0
Asian/Oriental	2 (1.6)	6 (10.3)
Black	4 (3.1)	2 (3.4)
Other	3 (2.3)	0
White/Caucasian	116 (90.6)	50 (86.2)
Ethnicity, n (%)		
Hispanic or Latino	17 (13.3)	8 (13.8)
Not Hispanic or Latino	110 (85.9)	50 (86.2)
Not reported/unknown	1 (0.8)	0
Weight, kg, mean (SD)	93.6 (19.7)	93.8 (16.4)
BMI, kg/m^2^, mean (SD)	32.6 (4.9)	33.0 (4.6)
Systolic blood pressure, mmHg, mean (SD)	130.0 (14.4)	134.9 (15.7)
Diastolic blood pressure, mmHg, mean (SD)	76.3 (9.8)	77.2 (9.2)
ASCVD (CHD, ischemic stroke, PAD), n (%)	47 (36.7)	24 (41.4)
HbA1c, %, mean (SD)	7.0 (0.9)	7.3 (0.8)
Fasting plasma glucose, mg/dL, mean (SD)	150.2 (39.2)	153.2 (41.1)
Individuals receiving insulin at baseline, n (%)	52 (40.6)	26 (44.8)
Baseline lipids, mean (SD)		
Non-HDL-C, mg/dL	170.3 (48.2)	166.3 (48.8)
mmol/L	4.41 (1.25)	4.31 (1.26)
LDL-C (beta-quantification), mg/dL	112.8 (43.7)	110.7 (42.1)
mmol/L	2.92 (1.13)	2.87 (1.09)
ApoB, mg/dL	109.3 (27.4)	108.1 (28.9)
LDL particle number, nmol/L	1497.3 (532.0)	1491.5 (545.1)
Lipoprotein(a), mg/dL, median (Q1:Q3)	18.0 (5.0:55.0)	9.5 (4.0:30.0)
TGs, mg/dL, median (Q1:Q3)	281.5 (245.0:369.0)	269.0 (232.0:328.0)
mmol/L	3.19 (2.77:4.17)	3.04 (2.62:3.71)
HDL-C, mg/dL	34.4 (6.2)	34.3 (5.9)
mmol/L	0.89 (0.16)	0.89 (0.15)

*ASCVD* atherosclerotic cardiovascular disease, *ApoB* apolipoprotein B, *BMI* body mass index, *CHD* coronary heart disease, *HbA1c* glycated hemoglobin, *HDL-C* high-density lipoprotein cholesterol, *ITT* intention-to-treat, *LDL* low-density lipoprotein, *LDL-C* low-density lipoprotein cholesterol, *PAD* peripheral artery disease, *SD* standard deviation, *TG* triglyceride

^a^Options included ezetimibe, fenofibrate, no additional lipid-lowering therapy, omega-3 fatty acid, and nicotinic acid

**Fig. 2:**
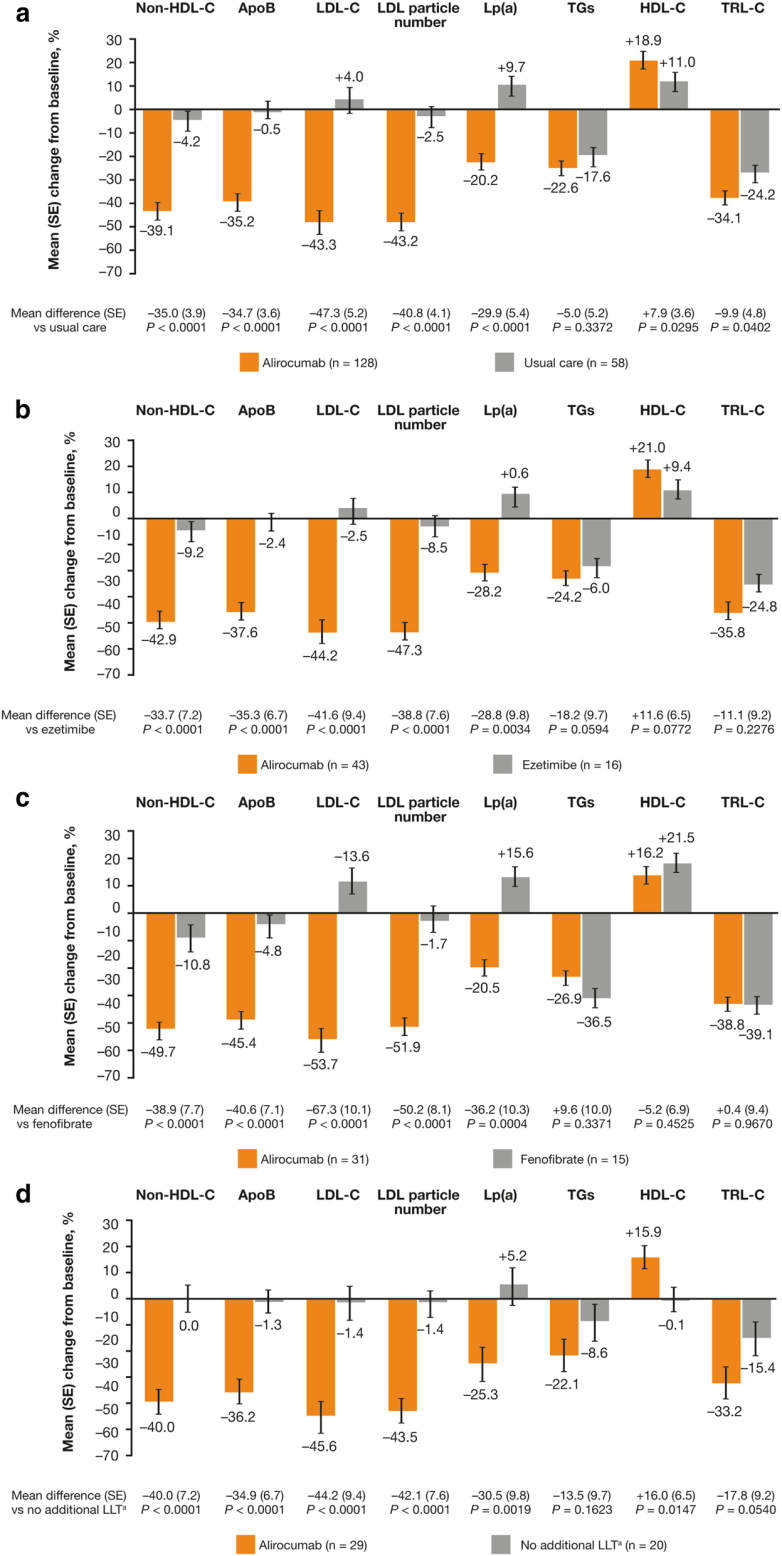
Percent change from baseline in LDL-C, non-HDL-C, ApoB, LDL particle number, Lp(a), TGs, HDL-C, and TRL-C at Week 24 with alirocumab versus usual care (panel **a**), ezetimibe (panel **b**), fenofibrate (panel **c**), and no LLT (panel **d**) (ITT population). ApoB, apolipoprotein B; HDL-C, high-density lipoprotein cholesterol; ITT, intention-to-treat; LDL, low-density lipoprotein; LDL-C, low-density lipoprotein cholesterol; LLT, lipid-lowering therapy; Lp(a), lipoprotein(a); SE, standard error; TG, triglyceride; TRL-C, triglyceride-rich lipoprotein cholesterol. Usual care options were selected by the investigator prior to stratified randomization to alirocumab or usual care. Usual care options included ezetimibe, fenofibrate, no additional LLT, omega-3 fatty acid, and nicotinic acid; due to low participant numbers, nicotinic acid and omega-3 fatty acid strata are not analyzed separately here. ^a^ No additional LLT on top of background maximally tolerated statin dose

Overall, alirocumab significantly reduced non-HDL-C [LS mean difference (SE): − 35.0% (3.9)] and ApoB [LS mean difference (SE): − 34.7% (3.6)], as well as LDL-C [LS mean difference (SE): − 47.3% (5.2)], LDL particle number [LS mean difference (SE) − 40.8% (4.1)], and Lp(a) [adjusted mean (SE]) − 29.9% (5.4)] from baseline to Week 24 versus usual care (all *P* < 0.0001; Fig. 2a). In addition, all comparisons for percentage change from baseline in non-HDL-C, ApoB, LDL-C, LDL particle number, and Lp(a) with alirocumab versus ezetimibe, fenofibrate, or no additional LLT were significant (*P* < 0.01; Fig. 2b–d, respectively).

Alirocumab reduced TGs to a greater extent than usual care overall, ezetimibe, or no LLT [adjusted mean difference (SE): − 5.0% (5.2) vs usual care; − 18.2% (9.7) vs ezetimibe; − 13.5% (9.7) vs no LLT]; however, mean TG reductions were greater with fenofibrate than alirocumab [adjusted mean difference (SE): 9.6% (10.0)]. *P*-values were not significant for any comparison (Fig. 2).

Overall, alirocumab significantly increased HDL-C compared with usual care [LS mean difference (SE): 7.9% (3.6); *P* < 0.05] and no LLT strata [LS mean difference (SE): 16.0% (6.5); *P* < 0.05]; however, LS mean difference (SE) with alirocumab versus ezetimibe or alirocumab versus fenofibrate was non-significant [11.6% (6.5) vs ezetimibe; − 5.2% (6.9) vs fenofibrate; Fig. 2]. The LS mean difference (SE) in TRL-C was significantly reduced with alirocumab versus usual care overall [− 9.9% (4.8); *P *= 0.0402; Fig. 2a]. However, *P*-values were not significant for alirocumab versus ezetimibe, fenofibrate, or no additional LLT (Fig. 2b–d).

**Fig. 3:**
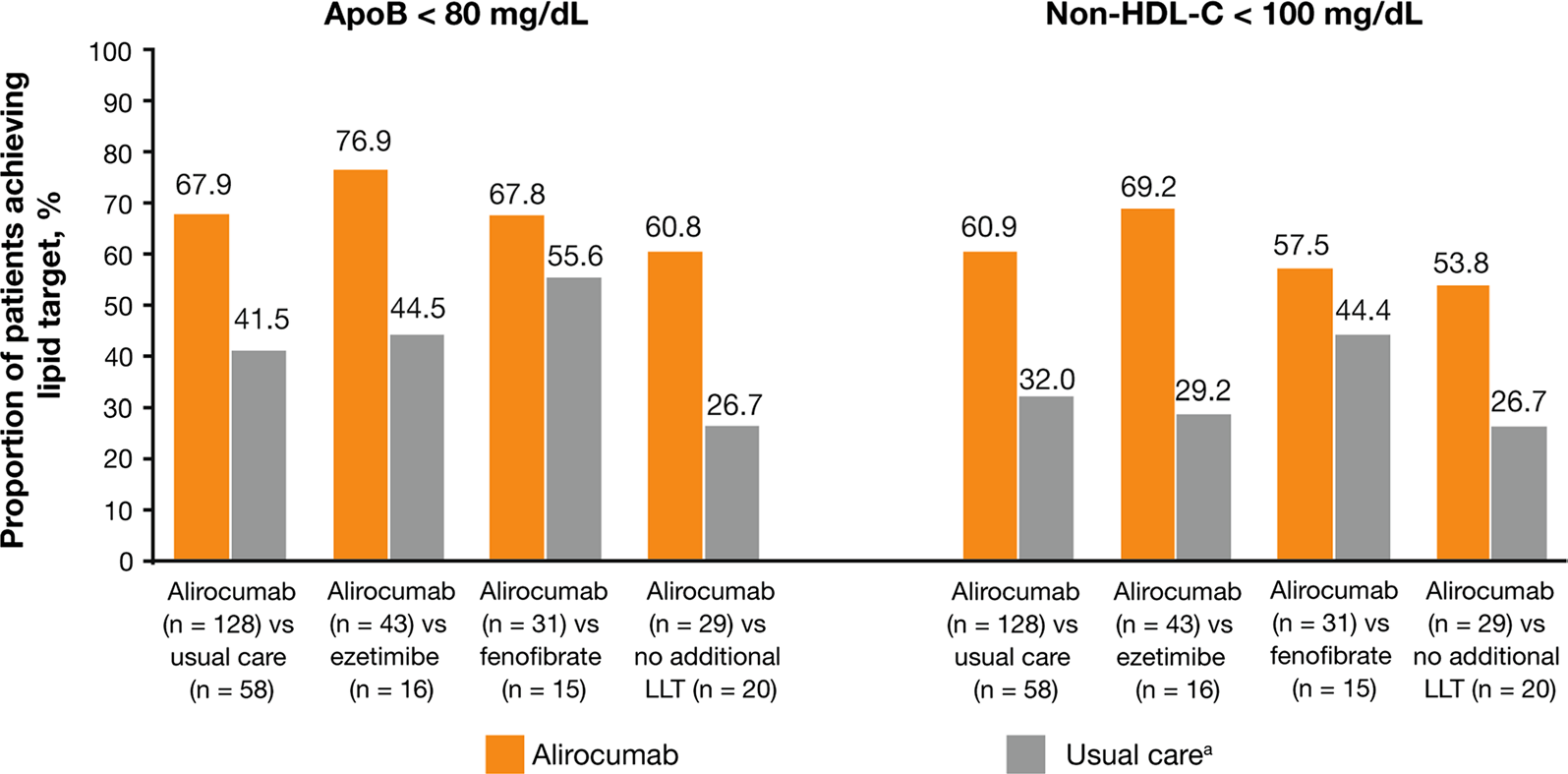
Proportion of individuals achieving ApoB < 80 mg/dL and non-HDL-C < 100 mg/dL for alirocumab versus usual care, alirocumab versus ezetimibe, alirocumab versus fenofibrate, and alirocumab versus no LLT (ITT population). ApoB, apolipoprotein B; HDL-C, high-density lipoprotein cholesterol; ITT, intention-to-treat; LLT, lipid-lowering therapy. ^a^ Usual care options included continuing on maximally tolerated dose of statins (or no statin if intolerant) with no additional LLT, or with the addition of ezetimibe, fenofibrate, omega-3 fatty acids, or nicotinic acid

ApoB < 80 mg/dL was achieved by 67.9% of alirocumab-treated individuals compared with 41.5% of individuals in the usual care group (Fig. 3). Similar trends were observed by strata (alirocumab vs ezetimibe: 76.9% vs 44.5%; alirocumab vs fenofibrate: 67.8% vs 55.6%; alirocumab vs no additional LLT: 60.8 vs 26.7%). In addition, non-HDL-C < 100 mg/dL was achieved in 60.9% of alirocumab-treated individuals compared with 32.0% of individuals in the usual care group. Likewise, similar trends were observed by strata (alirocumab vs ezetimibe: 69.2% vs 29.2%; alirocumab vs fenofibrate: 57.5% vs 44.4%; alirocumab vs no additional LLT: 53.8% and 26.7%).

The frequency of treatment-emergent adverse events (TEAEs) was similar in the alirocumab and usual care groups (67.2% vs 70.7%, respectively; Table 2). Treatment-emergent serious adverse events occurred in 6.3% and 5.2% of individuals receiving alirocumab and usual care, respectively. No deaths occurred in either treatment group. Discontinuations due to adverse events were similar in the alirocumab and usual care groups (3.1% vs 3.4%, respectively; Table 2). TEAEs occurring in ≥ 2% of individuals are also shown in Table 2. The most common TEAEs in the alirocumab group were urinary tract infection (8.6%), viral upper respiratory tract infection (5.5%), and influenza and diarrhea (both 4.7%). The most common TEAEs in the usual care group were bronchitis (8.6%), diarrhea (6.9%), and arthralgia (6.9%).

**Table 2:** Overview of TEAEs (safety population)

n (%)	Alirocumab (n = 128)	Usual care^a^ (n = 58)
Any TEAE	86 (67.2)	41 (70.7)
Any treatment-emergent SAE	8 (6.3)	3 (5.2)
Any TEAE leading to death	0	0
Any TEAE leading to permanent treatment discontinuation	4 (3.1)	2 (3.4)
TEAEs occurring in ≥ 2% of individuals (preferred level term)^b^		
Anemia	3 (2.3)	0
Arthralgia	2 (1.6)	4 (6.9)
Back pain	1 (0.8)	2 (3.4)
Bronchitis	1 (0.8)	5 (8.6)
Cellulitis	3 (2.3)	0
Cough	1 (0.8)	3 (5.2)
Diarrhea	6 (4.7)	4 (6.9)
Dizziness	2 (1.6)	2 (3.4)
Dyspnea	1 (0.8)	2 (3.4)
Fall	3 (2.3)	2 (3.4)
Fatigue	5 (3.9)	1 (1.7)
Headache	2 (1.6)	3 (5.2)
Hyperinsulinemic hypoglycemia	3 (2.3)	0
Hypoglycemia	2 (1.6)	2 (3.4)
Hypotension	1 (0.8)	2 (3.4)
Influenza	6 (4.7)	2 (3.4)
Injection-site bruising	3 (2.3)	0
Injection-site pruritus	4 (3.1)	0
Injection-site reaction	5 (3.9)	0
Muscle spasms	3 (2.3)	1 (1.7)
Musculoskeletal pain	4 (3.1)	2 (3.4)
Myalgia	3 (2.3)	1 (1.7)
Nausea	4 (3.1)	2 (3.4)
Osteoarthritis	0	2 (3.4)
Pain in extremity	1 (0.8)	2 (3.4)
Sinusitis	1 (0.8)	2 (3.4)
Type 2 diabetes mellitus	1 (0.8)	2 (3.4)
Upper respiratory tract infection	5 (3.9)	2 (3.4)
Urinary tract infection	11 (8.6)	2 (3.4)
Viral upper respiratory tract infection	7 (5.5)	1 (1.7)
Vitamin D deficiency	0	2 (3.4)

*SAE* serious adverse event, *TEAE* treatment-emergent adverse event

^a^Options included ezetimibe, fenofibrate, no additional lipid-lowering therapy, omega-3 fatty acid, and nicotinic acid

^b^Given in alphabetical order

Mean (SD) change from baseline at Week 24 in fasting plasma glucose was + 11.3 (51.5) mg/dL and + 2.9 (50.3) mg/dL, and in HbA1c was + 0.3 (0.7)% and + 0.3 (0.7)%, in the alirocumab and usual care groups, respectively. The number of antihyperglycemic agents being used was similar at baseline and Week 24 in both the alirocumab group [1.9 (1.0) and 2.0 (1.0), respectively] and the usual care group [2.0 (1.0) and 2.1 (1.0), respectively].

## Discussion

Individuals with T2DM are at increased risk of ASCVD [1], and mixed dyslipidemia further increases this risk [3, 18]. A recent analysis of 9593 statin-treated adults in the US National Health and Nutrition Examination Surveys found that the prevalence of TGs < 150, 150–199, and ≥ 200 mg/dL was 68.4%, 16.2%, and 15.4%, respectively [19]. In those on statin therapy with TGs ≥ 200 mg/dL, approximately half a million ASCVD events were estimated to occur in the next 10 years, with an estimated 10-year ASCVD risk score of 14.4%, compared to 11.3% for those with TGs < 150 mg/dL [19]. Furthermore, individuals with low HDL-C levels, despite receiving statin therapy, have been shown to have higher residual cardiovascular risk [20, 21]. There is therefore an opportunity for cardiovascular outcomes to be improved in individuals with T2DM and dyslipidemia who are receiving statin therapy.

Current approaches to reducing cardiovascular risk are tackling production of TG or ApoB particles [22, 23]. An alternative way to reduce residual risk is to reduce atherogenic lipoproteins. We tested this hypothesis in this subgroup of individuals with T2DM, elevated TGs, and low HDL-C. In these individuals, alirocumab significantly reduced LDL-C, non-HDL-C, ApoB, Lp(a), and LDL particle number compared with usual care. These results were comparable with the primary trial [14]; however, this analysis provides insight to the effects of alirocumab in the subgroup of individuals with high TG and low HDL-C despite statins, and who have higher residual cardiovascular risk than those without dyslipidemia. Most individuals receiving alirocumab achieved ApoB < 80 mg/dL and non-HDL-C < 100 mg/dL (67.9% and 60.9%, respectively). As these lipid parameters are associated with increased cardiovascular risk [2], the improvements observed with alirocumab may result in decreased cardiovascular risk. Similar findings have been obtained with evolocumab: the BANTING trial (NCT02739984) demonstrated that evolocumab significantly reduced LDL-C and non-HDL-C compared with placebo in adults with T2DM and hypercholesterolemia/dyslipidemia on a maximally tolerated oral dose of statin over 12 weeks [24].

Consistent with previous findings in participants with T2DM [14, 25], alirocumab resulted in non-significant TG reductions. These data confirm that blocking extra-cellular PCSK9 pathways with PCSK9 monoclonal antibodies does not affect hepatic ApoB production, and that the modest reduction in TGs is likely due to an increased uptake/catabolism of large very-low-density lipoprotein particles through the LDL receptor [26]. In previous studies with gemfibrozil in the Helsinki Heart Study [27] and fenofibrate in the ACCORD trial [15], TG lowering was generally not associated with overall cardiovascular benefit, but improvements were observed in subgroups with high TGs and low HDL-C (Helsinki Heart Study: TGs > 200 mg/dL, LDL-C/HDL-C ratio > 5.0; ACCORD: TGs ≥ 204 mg/dL, HDL-C ≤ 34 mg/dL).

This post hoc analysis provides useful data for comparison with several recently completed or ongoing cardiovascular outcome trials with similar thresholds for TGs and HDL-C. The REDUCE-IT trial demonstrated a reduction in cardiovascular events with 4 g of icosapent ethyl versus placebo (hazard ratio, 0.75; 95% confidence interval, 0.68–0.83; *P* < 0.001) over a median follow up of 4.9 years [17, 28]. In the ASCEND trial (NCT00135226), 1 g of eicosapentaenoic acid once daily did not reduce the risk of cardiovascular events versus placebo [29]. Other trials are currently ongoing with 4 g of omega-3 carboxylic acids (STRENGTH, NCT02104817) and pemafibrate (PROMINENT, NCT03071692).

Consistent with previous studies, alirocumab was well tolerated in individuals with T2DM [14, 25, 30, 31]. In addition, no clinically relevant effect of alirocumab on change in glycemic parameters or in use of antihyperglycemic agents was observed, in accordance with previous data [32]. A recent study in patients with stable CAD demonstrated that low PCSK9 plasma levels are associated with low HDL-C, metabolic syndrome, obesity, insulin resistance, and diabetes, and diffuse non-obstructive coronary atherosclerosis [33]. However, studies to date have not shown an association between PCSK9 inhibitors and low HDL-C or increased risk of diabetes, metabolic syndrome, or obesity [34–36]. Furthermore, similar safety findings were observed with alirocumab across BMI subgroups, with no difference in percentage change from baseline in body weight observed between alirocumab and control at Weeks 12, 24, and 52 [34]. However, studies of longer duration are required to further assess these potential effects.

This subgroup analysis was limited by the post-randomization nature of post hoc analyses, the relatively low number of participants, and the short duration of the study (24 weeks). In addition, comparisons were made with no adjustment on the type I error rate. Although DM-DYSLIPIDEMIA was not designed to assess cardiovascular outcomes, these findings are supported by the results of the ODYSSEY OUTCOMES cardiovascular outcomes trial [37]. A prespecified analysis of the ODYSSEY OUTCOMES trial showed that alirocumab treatment targeting LDL-C 25–50 mg/dL produced approximately twice the absolute reduction in cardiovascular events in individuals with diabetes as in those without diabetes. In addition, alirocumab treatment did not increase the risk of new-onset diabetes [31]. Since the objective of this post hoc analysis was to analyze a subgroup of patients with TGs > 200 mg/dL and low HDL-C, no analysis by stratification by baseline TG levels were conducted; however, in the primary paper, reduction in non-HDL-C was similar at TG thresholds of < 150, 150–< 200, and ≥ 200 mg/dL [14].

## Conclusions

In individuals with T2DM and mixed dyslipidemia, alirocumab significantly reduced LDL-C, non-HDL-C, ApoB, Lp(a), and LDL particle number, and significantly increased HDL-C, compared with usual care overall. Reduction with alirocumab in atherogenic lipids (ApoB and non-HDL) was greater than with ezetimibe, fenofibrate, or no additional LLT. In addition, alirocumab was effective for achieving target non-HDL-C and ApoB, compared with usual care, in this high cardiovascular risk subgroup population. Consistent with previous studies, alirocumab was generally well tolerated.

## Data Availability

Data sharing is not applicable to this article as it reports secondary analyses from primary data previously published, as cited in the reference list.

## Abbreviations

ApoB: Apolipoprotein B
ASCVD: Atherosclerotic cardiovascular disease
HbA1c: Glycated hemoglobin
HDL-C: High-density lipoprotein cholesterol
LDL: Low-density lipoprotein
LDL-C: Low-density lipoprotein cholesterol
LLT: Lipid-lowering therapy
Lp(a): Lipoprotein (a)
LS: Least squares
PCSK9: Proprotein convertase subtilisin/kexin type 9
SD: Standard deviation
SE: Standard error
T2DM: Type 2 diabetes mellitus
TEAE: Treatment-emergent adverse event
TG: Triglyceride
TRL: Triglyceride-rich lipoprotein
TRL-C: Triglyceride-rich lipoprotein cholesterol
